# The influence of artificial nucleus pulposus replacement on stress distribution in the cartilaginous endplate in a 3-dimensional finite element model of the lumbar intervertebral disc

**DOI:** 10.1097/MD.0000000000009149

**Published:** 2017-12-15

**Authors:** Yu Wang, Xiao-Dong Yi, Chun-De Li

**Affiliations:** Department of Orthopedics, Peking University First Hospital, Beijing, China.

**Keywords:** artificial nucleus pulposus, cartilaginous endplate, finite element analysis, intervertebral disc, stress distribution

## Abstract

**Objective:**

This study aimed to investigate the effects involved with the artificial nucleus pulposus (NP) replacement on stress distribution of the cartilaginous endplate (CEP) in a 3-dimensional lumbar intervertebral disc (IVD) model using a finite element (FE) analysis.

**Methods:**

A healthy male volunteer was recruited for the purposes of the study and a spiral computed tomography scan was subsequently conducted to obtain the data information in relation to the L_4/5_ motion segment. An FE model of the L_4/5_ motion segment constructed, on the basis of which degenerative IVD, IVD with NP removal, and IVD with NP replacement were in turn built. The stress distribution of the CEP and bulging of IVD were estimated using various motion states, including axial loading, forward flexion, backward extension, left axial rotation, and right axial rotation.

**Results:**

Under different motion states, the vertebral stress was higher in the degenerative IVD, the IVD with NP removal, and the IVD with NP replacement, in comparison to that of the normal IVD. Furthermore, a higher vertebral stress was detected in the degenerative IVD than the IVD with NP removal and the IVD with NP replacement. An even distribution of vertebral stress was observed in the IVD model with an artificial NP replacement, while the vertebral stress and bulging displacement were lower than after NP removal. Our findings provided confirmation that stress of the CEP was consistent with the vertebral stress.

**Conclusion:**

This study provided evidence suggesting that NP replacement, vertebral stress, and bulging displacement are lower than that of degenerative IVD and IVD with NP removal under different motion states.

## 1 Introduction

The complicated and unique structure of the intervertebral disc (IVD) allows for its delivery of exclusive properties, such as the ability to support the weight of the entire boy in addition to a wide variety of loadings and dynamic motions on the spinal cord.^[1]^ The IVD is the largest avascular structures in the human body, which undergoes more extensive changes than any other structure with increases in both age and degeneration.^[2]^ The IVD is primarily comprised of 3 distinct anatomical parts: the central nucleus pulposus (NP), the annulus fibrosus, and 2 cartilaginous endplate (CEP) in the upper and lower sides.^[3]^

The CEP is a layer of hyaline cartilage approximately 600 μm thick positioned between the NP and the vertebral endplates. The CEP has distinct properties from the vertebral endplates, which are largely composed of cortical bone.^[4]^ The CEP can function as a barrier between the vertebral bone and the pressurized NP, while also providing an avenue for the transportation of nutrients into the discs from adjacent blood vessels.^[5]^ IVD degeneration can in some instances be accompanied by progressively sclerotic CEP, which may lose contact with vasculature, and exhibit a reduction in permeability.^[6]^ Recent years have seen cell transplantation for degenerated IVD continue to progressively be viewed as a favorable procedure, but studies have shown that it must be performed through the annulus fibrosus. However, research has indicated that the NP can be approached through the CEP via pedicle with no effects on the neural foramina or the spinal canal, which could be an alternative approach to reach the NP, simultaneously without the disruption of annulus fibrosus.^[7,8]^ At present, it is thought that the mechanical stresses acting upon the IVD are representing an important factor in the pain experienced.^[9]^ Furthermore, previous studies have highlighted that altered stress distribution could potentially lead to damage of the underlying vertebrae as well as bone remodeling.^[10]^ Interestingly, Li et al^[11]^ revealed during their study that the changes observed in relation to the stress distribution of IVD may result in the structure of IVD becoming increasingly unstable, which could result in the occurrence of intervertebral cleft with the gradual filling with gas in the event of compression, thus leading to the exacerbation of lower back pain and degeneration of the IVD.

In the past few years, clinical debates and accumulating arguments have stressed the importance of the CEP and NP in the treatment of IVD degeneration.^[12–14]^ A recent study has been conducted into the various modeling of lumbar IVD mechanics under different physical conditions, in order to better elucidate the functions of the native disc tissues under various loading conditions.^[15–17]^ However, insufficient literature is available emphasizing the use of modeling to explore the behavior of NP replacement. Thus, during this study it was hypothesized that the NP implant cavity and modulus could influence the load distribution of the IVD.^[3]^ However, little evidence based on experimental results has been able to demonstrate the stress state of the IVD or the CEP on the overall mechanical behavior of the disc. Therefore, the central objective of the present study was to investigate the effects of artificial NP replacement in lumber IVD on the stress distribution of CEP based on a finite element (FE) analysis.

## 2 Materials and methods

### 2.1 Ethics statement

The experiment was conducted under the approval of the Ethics Committee of the Peking University First Hospital. All participants in the study were provided with informed consent documentation.

### 2.2 Establishment of the lumbar vertebrae and L_4/5_ motion segment models

**Figure 1:**
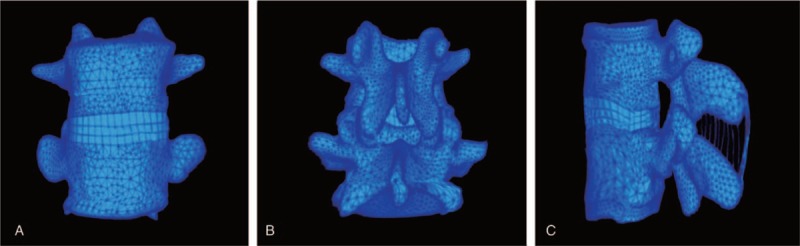
The 3-dimensional finite element model of L_4/5_ motion segment. (A) Anterior view of the L_4/5_ motion segment model; (B) posterior view of the L_4/5_ motion segment model; (C) lateral view of the L_4/5_ motion segment model.

A 22-year-old healthy male volunteer, with a height of 169 cm, weighing 63 kg was selected for the study. An X-ray examination was conducted to ensure that the volunteer was free of any pathological deformations such as spinal damage, degeneration, and congenital malformation. Modic classification on magnetic resonance imaging (MRI) was employed which indicated that the L_4/5_ IVD was free of any CEP degeneration. Spiral computed tomography (CT) was adopted to scan the lumbar vertebrae of the volunteer. A 1 mm-thick image of a serial cross-section was acquired and then input into the Mimics15.0 software. The threshold value of the image was adjusted to 462 to 2676 Hounsfield unit and all the required bone structure segments of lumbar vertebrae were roughly acquired by use of region growing. Next, detailed modifications were performed on a step-by-step basis in order to outline the bone structures. XY-plane was defined as the L4 superior vertebral endplate. The sagittal plane represented the Y-axis (anterior and posterior), while the coronal plane was representative of the X-axis, and the vertical direction as the Z-axis (top to bottom). A 3-dimensional (3D) model of the lumbar vertebrae L_4/5_ segment is shown in Fig. 1. The shape of the model was confirmed to be of high quality in the visual effect, and no difference was observed in relation to the actual skeleton. The relationship between the inner structures in each component could be observed in different planes. Observations of the rotation angle and zoom could be viewed arbitrarily; thus, the L4/5 segment model can be used for an FE analysis. The model was divided into 58,595 units and 110,827 nodes, and the specific physical parameters, unit types, and related materials of the lumbar vertebrae are shown in Tables 1 and 2. Next, the data of the 3D model were exported to the GeomagicStudio10.0 reverse engineering software for the generation of an entity model of the lumbar vertebrae.^[18]^ All materials were simplified to isotropic materials as well as for the description of the elastic modulus and Poisson ratio. The Poisson ratio of the NP was defined as 0.4999, close to 0.5. The ligament was a material that could only be subjected to a tensile load. The modulus of elasticity of the matrix was adopted with smaller loads in lumbar spine, deformation, and fiber action of annulus fibrosus.^[19,20]^ The assembled entity model was input into the FE analysis software Ansys12.0 for mesh generation. In light of the anatomic sites and morphologies, CEP, annulus fibrosus, and NP were established. Afterward, the ligaments were added using a Link unit, including anterior longitudinal ligament, posterior longitudinal ligament, ligamentum flavum, interspinous ligament, supraspinous ligament, capsular ligament, and transverse ligament.^[21]^ The NP was modeled as one in under incompressible hydrostatic pressure status. Articular facets were designed as uneven oval surfaces to simulate the physiological morphology. The establishment of all the structures was used to assist in the construction of various 3D models using Ansys FE analysis software.

**Table 1:**
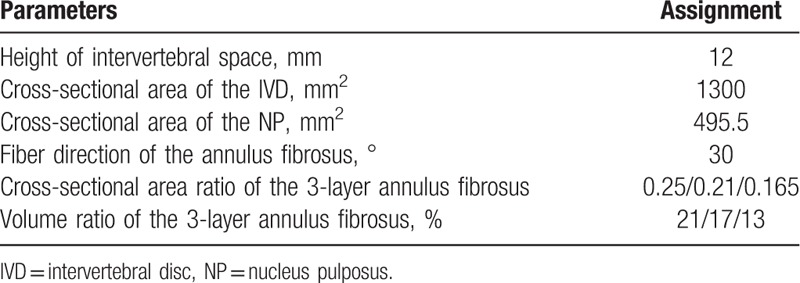
Physical parameters of the vertebral body.

**Table 2:**
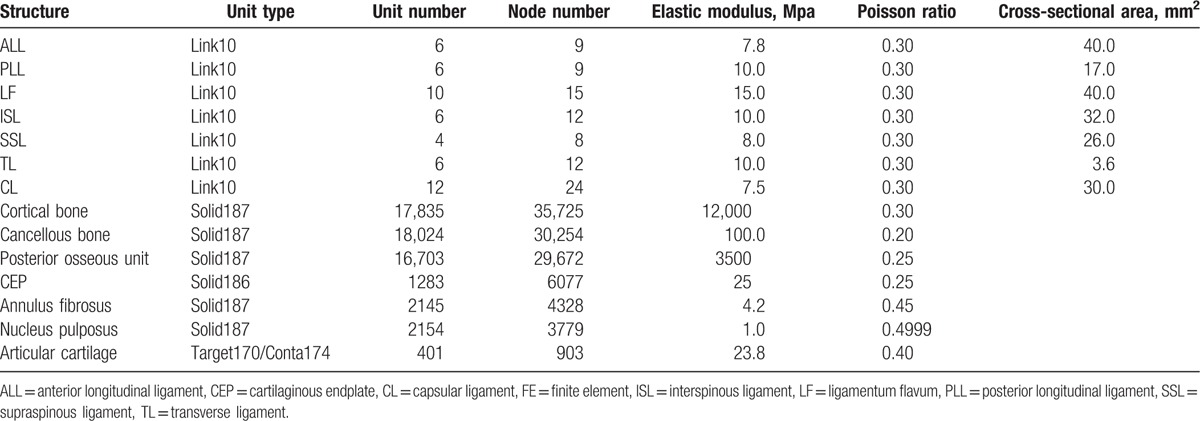
Unit types and material properties of the L_4/5_ segment FE model.

### 2.3 Establishment of the normal IVD, degenerative IVD, IVD with NP removal, and IVD with NP replacement

The normal IVD was a 3D model comprising of annulus fibrosus, CEP, and NP, with identical properties of the biomaterials of the L_4/5_ segment. The NP in the model was in hydrostatic pressure status and the pressure was 1.5 times of the axial compressive load. The height of the IVD was 12.00 mm and the cross-sectional area of the IVD was 1300 mm^2^. The cross-sectional area of the NP was 495.5 mm^2^; the fiber direction of the annulus fibrosus was 30°. The cross-sectional area ratio of the 3-layer annulus fibrosus was 0.25/0.21/0.165 and the volume ratio was 21%/17%/13%, respectively. The elastic modulus of the NP was 1 Mpa, the Poisson ratio was 0.49, and the shear modulus was 0.338 Mpa; the elastic modulus of the annulus fibrosus was 2.56 Mpa, Poisson ratio was 0.40, and the shear modulus was 0.914 Mpa.

The degenerative IVD was constructed based on the FE model of a normal L_4/5_ segment. The biological properties of the CEP were identical to the L_4/5_ motion segment. The elastic modulus of the NP was 1.66 Mpa, Poisson ratio was 0.40, and the shear modulus was 0.593 Mpa; the elastic modulus of the annulus fibrosus was 12.99 Mpa, Poisson ratio was 0.35, and the shear modulus was 4.55 Mpa.

The IVD with NP removal was composed of an annulus fibrosus, CEP, and NP, all with identical biological properties to the 3D model of the L_4/5_ segment. The model was constructed using simulation of the anterior lumbar NP removal, and the internal pressure of the NP was set to zero.

The IVD with NP replacement was built on the basis of the 3D FE model of a normal L_4/5_ segment, by simulating the anterior artificial lumbar IVD replacement. The NP of a normal L_4/5_ segment was replaced with that of an SMH artificial lumbar IVD, while the fiber and cellular matrix of the annulus fibrosus remained the same.

### 2.4 Application of vertical load

The vertical load opposite to the Z-axis direction was applied to the top surface of the L_4_ vertebral body, which had also been evenly distributed at 40 points on the top surface. According to the stress distribution characteristics of a human vertebra that cortical bone bears and passes, the main loads and the cancellous bone bears fewer loads comparatively speaking. The load distribution was set at a ratio of 3:1 on the cortical bone and cancellous bone. The specific loads were determined based on the experimental requirements. Static structure analysis procedures of higher order were executed for the data operation. An AEDIT order was executed in a sequential manner in order to adequately construct images. Following the modification of the image data, an FE model of a human L_4/5_ motion segment was successfully established.

### 2.5 Verification of the lumbar IVD model

The vertebral stress distribution under loading was observed in order to verify the biofidelity of the L_4/5_ motion segment model. The obtained evaluation results were compared with previous experiments. Under a vertical compressive load of 1000 to 6000 N (increase 1000 N for each time), with the average value of Z-axial displacement at all points on the top surface of the L_4_ vertebral body as the vertical Z-axial displacement of the L_4/5_ motion segment, an axial compressive stress-axial displacement curve was drawn. The experimental results of Lu et al,^[22]^ Brown et al,^[23]^ and Markolf et al^[24]^ were used as references for verification purposes in relation to whether the internal stress distribution of the IVD was in accordance with the basic biomechanical theories of IVD. Next, under a vertical compressive load of 1000 to 6000 N (increase 1000 N for each time), the average value of axial stress at all points on the top surface of the L_4_ vertebral body was used as the standard for the assessment of the axial compressive stress of the vertebral body. The stress value was the central point on the central layer of the IVD as the standard to evaluate the internal axial compressive stress of the IVD. The average values of the Y-axial displacements at the posterior lateral point of all layers of IVD were set as the standard for the evaluation of the bulging displacement of the posterior lateral annulus fibrosus of the IVD. The axial compressive stress of the vertebral body–the bulging displacement of the posterior lateral annulus fibrosus of the IVD curve was then drawn, as well as an axial compressive stress of the vertebral body–the internal stress of the IVD curve, which were compared with the experimental curves of Lu et al,^[22]^ Markolf et al,^[24]^ Rolander et al,^[25]^ and Nachemson.^[26]^ An axial compressive load of 500 N was applied to the upper surface of the L4 vertebral body to simulate the weight of the upper portion of the human body. All the nodes on the upper surface of the L4 vertebral body were coupled to the neutral point. In accordance with the right-hand rule, a torque of 7.5 Nm was applied at the neutral point, and forward flexion, backward extension, left axial rotation, and right axial rotation were all stimulated, respectively, in a simultaneous manner. All the nodes on the lower surface of the L5 vertebral body were fixed to limit all degrees of freedom.^[27,28]^

### 2.6 Three-dimensional FE analysis

The above L_4/5_ motion segment model was replaced by the corresponding models of the normal IVD, degenerative IVD, IVD with NP removal, and IVD with NP replacement. The L_5_ vertebral body was fixed and an axial compressive load of 500 N was applied on the upper surface of the L_4_ vertebral body to simulate the weight of the upper human body. Meanwhile, extra loads were added in an evenly distributed manner to the surface of the CEP. Moreover, all the nodes on the upper surface of the L_4_ vertebral body were coupled to the neutral point. In accordance with the right-hand rule, a torque of 7.5 Nm was applied at the neutral point, and forward flexion, backward extension, left axial rotation, and right axial rotation were stimulated, respectively, simultaneous manner; all the nodes on the lower surface of the L_5_ vertebral body were fixed to limit all degrees of freedom.^[29]^ In the end, the peak values of the stress on the CEP were calculated and the stress distribution was recorded. The bulging of the L_5_ vertebral body was recorded and analyzed under the same loading conditions in each group.

## 3 Results

### 3.1 Verification of the 3D FE model of L_4/5_ segment

**Figure 2:**
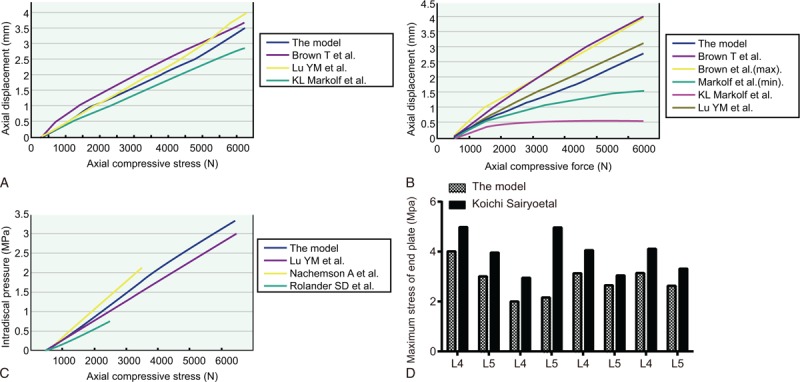
The analysis of the L_4/5_ segment FE model under different axial compressive loading. (A) The axial compressive stress-axial displacement curve; (B) the vertebral axial compressive stress-bulging displacement of the posterior lateral IVD curve; (C) the vertebral axial compressive stress-internal stress of the IVD curve; (D) maximum equivalent stress of the L_4/5_ segment under different axial compressive loading. FE = finite element, IVD = intervertebral disc.

The axial displacement of the vertebral body increased in a linear manner with the increase of loading. The curve fell between the mechanical curves of previously conducted experiments by Markolf et al^[24]^ and Brown et al,^[23]^ and coincided with the curve from the study of Lu et al^[22]^ (Fig. 2A). The bulging displacement of the posterior lateral IVD also presented a linearly positive correlation with the increase of loading. The curve fell between the corresponding curves of the experiments conducted by Lu et al^[22]^ and Brown et al^[23]^ (Fig. 2B). The results verified that the flexibility and stiffness of the IVD in the established FE model were similar to that of actual human vertebrae. The results were in accordance with the basic rules of load transfer: stress bore by vertebra and the internal stress of IVD that occur in turn with increased loading. Moreover, the test values were similar to the data of previous experiments conducted by Lu et al,^[22]^ Rolander et al,^[25]^ and Nachemson^[26]^ (Fig. 2C). Lumbar vertebrae stress was primarily distributed throughout the areas with high elastic modulus, such as in cortical bone. Stress on the cancellous bone was distributed in the shape of a Chinese character “I” under vertical loading, which was identical to that of a 3D model of lumbar vertebral cancellous bone, which was also in accordance with the previous FE analysis results. The maximum equivalent stress of the CEP was in accordance with the results of Sairyo et al^[30]^ (Fig. 2D). The maximum stress of the L_5_ endplate was greater than that of the L_4_ endplate when the lumbar vertebra was backward extended. The maximum stress of the L_4_ endplate was greater than that of the L_5_ endplate in the event of forward flexion and rotation. The aforementioned results provided evidence that allowed for verification, which the load capacity of the experimental model was similar to that of an actual human vertebra. The test conducted with a normal IVD model revealed that stress on IVD was mainly transferred outward from the NP. The annulus fibrosus bore corresponding tensile stress, which was relatively concentrated on the posterior lateral annulus fibrosus.

### 3.2 Stress distribution on the vertebral body

**Figure 3:**
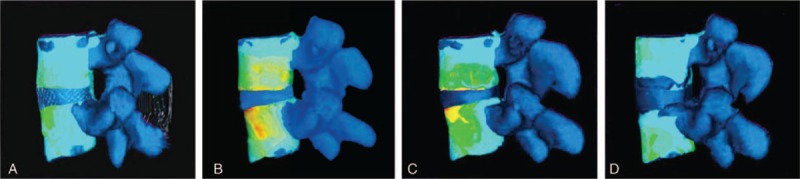
The stress distribution of the vertebral body under the axial load of 500 N in the 4 models. (A) Normal IVD; (B) degenerative IVD; (C) IVD with NP removal; (D) IVD with NP replacement; the color form blue to red represented stress rise. IVD = intervertebral disc, NP = nucleus pulposus.

The vertebral stress was differently distributed in different motion states: stress was distributed on the anterior part of the vertebral body during forward flexion, on the posterior part of the vertebral body during backward extension, unevenly distributed during axial compression, on the right side of the vertebral body during right axial rotation, and on the left side of the vertebral body during left axial rotation. Under axial compression, the stress distributions on the vertebral surface in the 4 models were shown in Fig. 3.

### 3.3 Stress of the CEP in the 4 FE models under different motion states

**Figure 4:**
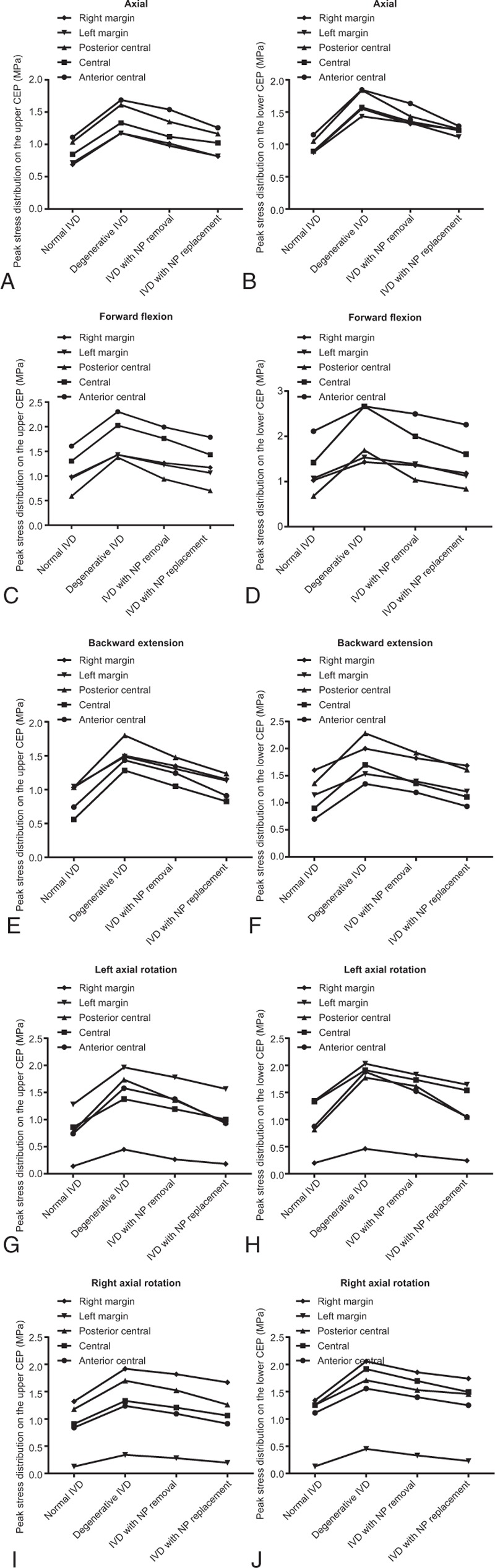
Peak stress distribution in upper and lower endplate of FE models in each group (MPa). (A) Peak stress distribution in the upper endplate (MPa)—anterior and middle parts; (B) peak stress distribution in the upper endplate (MPa)—middle parts; (C) peak stress distribution in the upper endplate (MPa)—posterior and middle parts; (D) peak stress distribution in the upper endplate (MPa)—left margin; (E) peak stress distribution in the upper endplate (MPa)—right margin; (F) peak stress distribution in the lower endplate (MPa)—anterior and middle parts; (G) peak stress distribution in the lower endplate (MPa)—middle parts; (H) peak stress distribution in the lower endplate (MPa)—posterior and middle parts; (I) peak stress distribution in the lower endplate (MPa)—left margin; (J) peak stress distribution in the lower endplate (MPa)—right margin. FE = finite element.

Representative nodes from the central part, left and right lateral margin, and posterior and anterior central parts on the L_4_ and L_5_ vertebrae were selected in the 4 models. The stress at those nodes was subsequently calculated. The stress of the upper and lower CEP in the 4 models under different motion states are shown in Fig. 4. When compared with the normal IVD, the stress level of the degenerative IVD, the IVD with NP removal, and the IVD with NP replacement under axial compression increased to 64.63%, 41.37%, and 20.69%, respectively; the stress level of the other 3 models increased to 57.88%, 31.56%, and 12.07% under forward flexion; increases to 61.69%, 39.47%, and 16.66% was observed during backward extension, while under left axial rotation, increases to 81.19%, 55.59%, and 21.43% was found. In the case of under right axial rotation, increases to 50.21%, 34.55%, and 19.10% was detected. The stress level was higher in the degenerative IVD than the IVD with NP removal and the IVD with NP replacement. The stress level was evenly distributed in the IVD with NP replacement, and it was significantly lower than in that of the IVD with NP removal. The results obtained were consistent with the study of Zhao et al.^[31]^

### 3.4 Bulging of the IVD in the 4 FE models under different motion states

**Figure 5:**
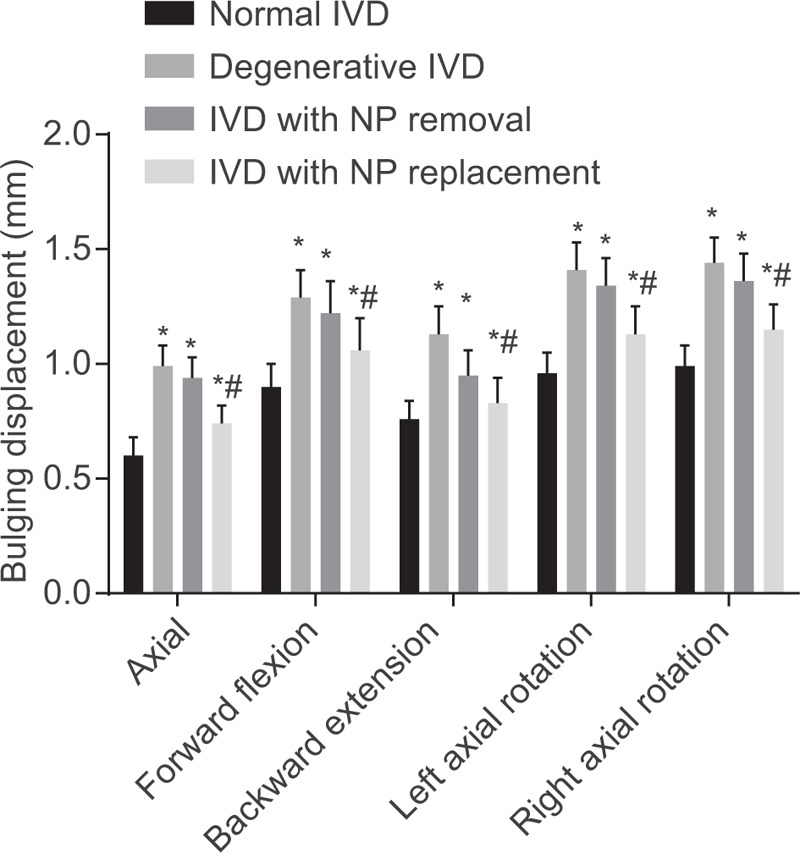
Bulging displacement of FE models in each group. “∗” presents the comparison of the results of the normal IVD group, *P* < .05; “#” presents the comparison of the results of the IVD with NP removal group, *P* < .05. FE = finite element, IVD = intervertebral disc.

Radial bulging of the IVD was exhibited by the compression side under different motion states. In comparison to the normal IVD, the bulging displacement increased in the degenerative IVD, the IVD with NP removal. The bulging displacement decreased in the IVD with NP replacement compared with the IVD with NP removal, suggesting that an artificial NP replacement could correct the biomechanical disorder after NP removal (Fig. 5).

## 4 Discussion

NP replacement is rapidly emerging as a popular treatment approach for degeneration of the lumbar IVD.^[32–34]^ However, little is understood concerning the relationship between the properties of the NP replacement and the stress distribution of the disc or the CEP. Therefore, the central objective of the present study was designed to explore the effects of NP replacement in lumber IVD on the stress distribution of CEP based upon FE analysis. The results obtained during the study indicated that after an NP replacement, the lumber IVD exhibits higher stress levels than in the case of normal IVD, and is maintained at an even load distribution under various states of movement and significantly lower in comparison to degenerative IVD and IVD with NP removal.

Initially, our experiment revealed the stress distribution in the degenerative IVD, the IVD with NP removal, and the IVD with NP replacement to have significantly increased. Based on our established model, it was confirmed that after NP removal and NP replacement, stress was reduced, compared to that of degenerative IVD. This finding was consistent with the study of Zhao et al, suggesting that lumbar IVD degeneration as having distinct effects on the stress distribution of the endplate.^[31]^ Previous reports have indicated that early IVD degeneration is characterized by a loss of water and proteoglycans in the NP, the internal region in the disc, causing decreased intradiskal pressure (over 30% reduction), followed by mechanical instabilities. The reduction of intradiskal pressure leads to decreased disc height and aberrant stress distributions in the annulus fibrosus.^[35]^ According to the study of Adams and Roughley,^[36]^ excessive mechanical loading causes a disc to degenerate, as well as the combinations of compression, torsion, and other factors can lead to the major structural features of disc degeneration, including endplate defects, radial fissures, and radial bulging. Previous evidence assessing the role of the NP in biomechanical behavior in the disc, highlighted that the removal of the NP may result in enhanced displacement and that the more NP removed, the greater amount of displacement.^[37]^ The experiment of Dolan et al concluded that a loss of NP pressure, deriving from endplates bulging into surrounding vertebrae, or from dehydration and desiccation, is capable of generating stress concentration to the endplates and subchondral bone trabeculae, which can lead to the occurrence of microfractures, subsequently, resulting in severe biomechanical consequences.^[38]^ As a thin layer of hyaline cartilage between the disc and the vertebral body, the CEP is the main pathway for nutrient transport to the avascular disc and for metabolite transport away from the disc, thus CEP degeneration may lead to or accelerate disc degenerative change.^[39]^ Based on current literature, in the lower lumbar vertebral bodies, the CEP has been reported to be an essential factor in the maintenance of the structural and functional integrity of local structures.^[40]^ CEP may become sclerotic, lose vasculature contact, and exhibit decreased permeability with IVD degeneration.^[6]^ In this circumstance, further studies of micro-CT in a pig model demonstrated an association between the permeability and bone marrow channel numbers in the vertebral endplate surface, which have also been confirmed to sharing a relationship with the mechanical loading distribution.^[41,42]^

The role of NP replacement is to restore segmental spinal biomechanics and normal loads to the diseased level, and slow adjacent level degeneration.^[43]^ After NP replacement, the vertebral stress is differently distributed under different motion states: stress is distributed on the anterior part of the vertebral body during forward flexion, on the posterior part of the vertebral body during backward extension, unevenly distributed during compression, and on the corresponding side of the vertebral body during axial rotation. The NP osmotic pressure can act as load supporter in compression and place the adjacent annulus fibrosus into circumferential tension, and in the setting of IVD degeneration, the NP proteoglycan content is reduced with decreasing pressure and modifying the overall disc load support, leading to increased range of motion in the axial loading.^[44]^ During the research conducted by Cannella et al, it was hypothesized that through the neutral zone, the initial loading of the IVD is dependent on the intradiscal pressure conferred by the nucleus to make the annulus fibrosus tense. Cannella et al^[37]^ also revealed that the compressive mechanics are predictable of the bending and torsional mechanics. Arthur et al^[45]^ reported that through the control of the NP replacement material implantation volume and pressure, compressive, bending, as well as torsional stability are restored to the disc. Evidence has suggested that by applying an NP replacement device of a compressive modulus ranging between 50 and 1500 kPa, restoration of lumbar IVD mechanics can be achieved, demonstrating some flexibility in compressive modulus of the given implant material to interface with the disc so as to stabilize the compressive mechanics.^[3]^ It is also implied that IVD instability can be rehabilitated by volume filling of the NP through application of the hydrogel device,^[35]^ which is consistent with our findings. Moreover, a previous study went on to highlight FE analyses as a promising tool in clinical diagnosis and plays an essential role in optimizing individual therapy in the protrusion of IVD, a 3D FE model of the lumbar is based upon the information directly derived from scan images such as CTs and MRIs. Furthermore, the results of FE analysis can indicate the stress and strain distribution of the spine.^[46]^ In the study of Yu et al,^[47]^ it was revealed that under different physiological loadings, the FE model is validated and applied to observe the motion and stress of the vertebrae. At present, the material characteristics of the artificial NP are different from those of the normal IVD that is composed with complex anatomical structure, and its biomechanical properties also show complex characteristics with anatomical and histopathological changes, Therefore, deeper fundamental theoretical research and large sample size were needed in the future research.

In conclusion, our study demonstrates that the crucial role played by the NP in relation to the biomechanical properties of the IVD through the analysis of the stress distribution under different motion sites of cartilage endplate and the influence of stress changes and cartilage degeneration on stress distribution. The stress distribution of CEP varied significantly after NP removal, and the transmission load almost disappeared in the NP. Our findings suggested that NP replacement can partly improve the transmission load, but may well cause the stress that was concentrated on the replacement area, which may lead to long-term adverse effects. Therefore, an NP replacement may serve as a practical treatment for disc diseases. The biological, histological, biochemical, and biomechanical characteristics of the IVD need to be further understood to provide basic data for the development of clinical treatment strategy and the reconstruction of the IVD joint function.
